# Ecosystem consequences of introducing plant growth promoting rhizobacteria to managed systems and potential legacy effects

**DOI:** 10.1111/nph.18010

**Published:** 2022-03-15

**Authors:** Jessica A. M. Moore, Paul E. Abraham, Joshua K. Michener, Wellington Muchero, Melissa A. Cregger

**Affiliations:** ^1^ Biosciences Division Oak Ridge National Laboratory 1 Bethel Valley Rd Oak Ridge TN 37830 USA

**Keywords:** agroecology, biostimulant, invasion ecology, microbiome, plant growth promoting bacteria, plant–microbe interactions

## Abstract

The rapidly growing industry of crop biostimulants leverages the application of plant growth promoting rhizobacteria (PGPR) to promote plant growth and health. However, introducing nonnative rhizobacteria may impact other aspects of ecosystem functioning and have legacy effects; these potential consequences are largely unexplored. Nontarget consequences of PGPR may include changes in resident microbiomes, nutrient cycling, pollinator services, functioning of other herbivores, disease suppression, and organic matter persistence. Importantly, we lack knowledge of whether these ecosystem effects may manifest in adjacent ecosystems. The introduced PGPR can leave a functional legacy whether they persist in the community or not. Legacy effects include shifts in resident microbiomes and their temporal dynamics, horizontal transfer of genes from the PGPR to resident taxa, and changes in resident functional groups and interaction networks. Ecosystem functions may be affected by legacies PGPR leave following niche construction, such as when PGPR alter soil pH that in turn alters biogeochemical cycling rates. Here, we highlight new research directions to elucidate how introduced PGPR impact resident microbiomes and ecosystem functions and their capacity for legacy effects.

## Introduction

Introduced species affect ecosystems in unpredictable ways because they are a new variable in a complex ecological network of existing interactions. Like other introduced species, plant growth promoting rhizobacteria (PGPR) have unpredictable ecosystem consequences and legacy effects; these outcomes are a current knowledge gap. Commercially available crop biostimulants sometimes contain PGPR that directly or indirectly increase plant productivity or crop yield, stimulate nutrient uptake, improve nutrient cycling efficiency, reduce pathogen loads, and increase plant tolerance to abiotic stress (Brown & Saa, 2015; Deng *et al*., 2019). Beyond the host plant effects, biostimulant PGPR may irreversibly shift communities and ecosystem functions to new states (Zheng *et al*., 2018), and therefore we urge future research in these critical knowledge gaps.

**Fig. 1:**
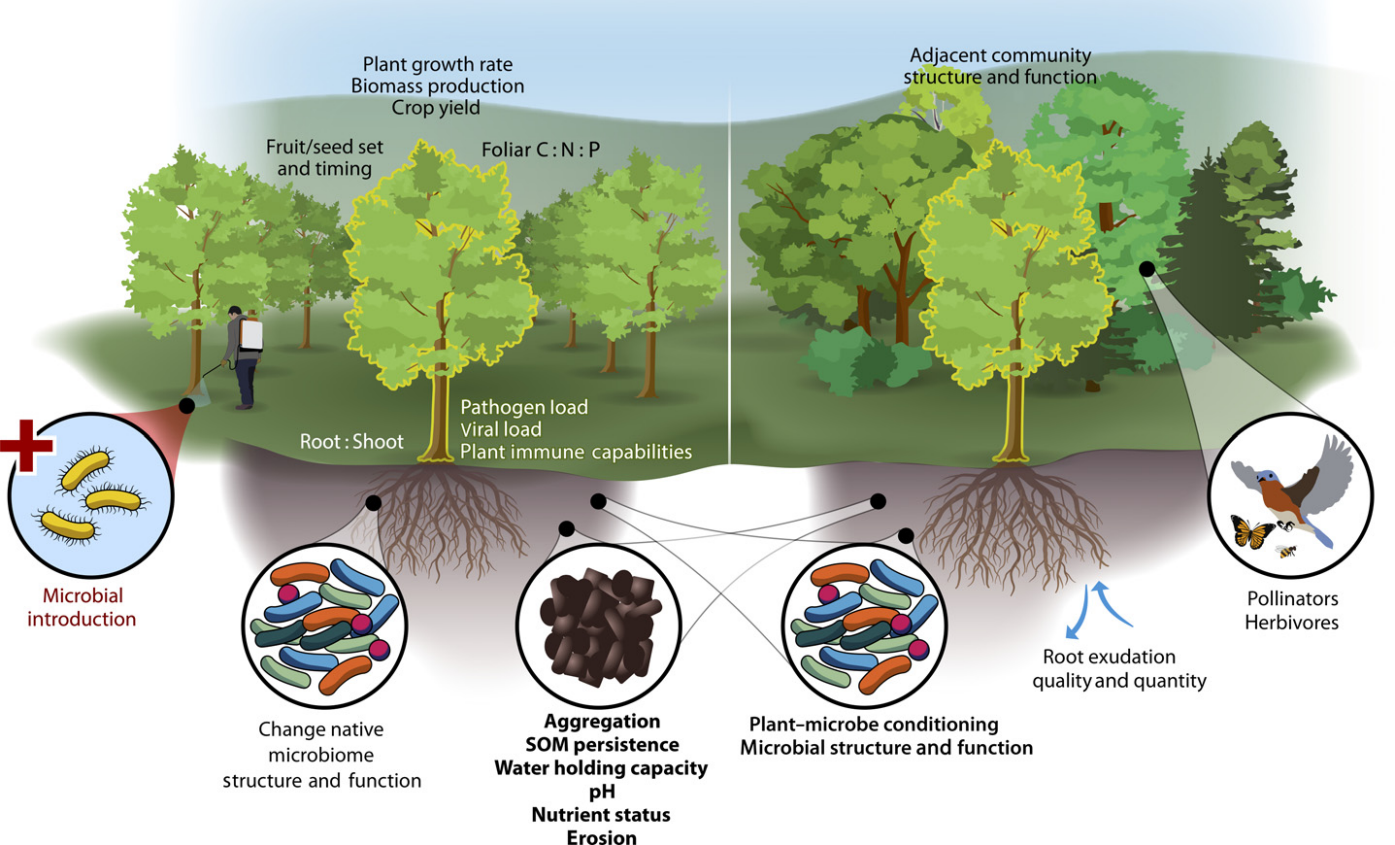
The long‐term consequences of inoculating soils with plant growth promoting rhizobacteria (PGPR) could lead to unintentional changes in soil properties, biotic communities and interactions, and ecosystem functions. Inoculating soils has the immediate intended effect of increasing plant productivity, shifting resource allocation to promote fruiting or crop yield, or reducing pathogen infections. Introducing PGPR could have further effects such as changing the resident microbiome structure and function and altering plant root exudate quantity and chemistry, which would cascade to influence soil aggregation, soil organic matter (SOM) persistence, pH, nutrient status, erodibility, and water holding capacity. Research is needed on consequences for herbivore and pollinator communities that depend on a crop’s time of fruiting or seed set or the duration of the growing season. An outstanding knowledge gap, due to lack of field‐based research, is whether PGPR inoculants disperse to adjacent ecosystems and their potential legacy effects. Artist credit: Andrew Sproles, Creative Services, Communications Division, Oak Ridge National Laboratory (TN, USA).

While PGPR effects on host plants have been long studied, there is a dearth in research on their influence on ecosystem functions or legacies left in the resident community. A keyword search in the Web of Science found 313 articles describing biostimulants containing PGPR published since 1998 (‘biostimulant’ AND (‘rhizobacteria’ OR ‘bacteria’ OR ‘microb*’), webofscience.com). Interest in this research topic has expanded in recent years. Before 2015, fewer than 10 articles per year were published on this topic; the count jumped to 50+ per year since 2019. Despite increased interest in PGPR, only 12 of the 313 articles included the search term ‘ecosystem’ and one article included ‘legacy’. Here, we highlight potential effects of PGPR species introductions that may extend beyond the host plant to ecosystem functions that may persist longer than the PGPR populations (Fig. 1). We propose new research directions necessary to improve predictions of the effects of biostimulants containing PGPR on ecosystem functions and their legacies (Box 1).

Box 1  

Here, we describe seven open research questions regarding PGPR inoculants, their influence on ecosystem functions, and their legacies. These questions are critical for improving the predictability of consequences of introducing PGPR. While some research into these questions has begun, these questions warrant further investigation because complete understanding of the mechanisms or relevant scale is still lacking, especially under field conditions.

1. *How do PGPR affect macronutrient and micronutrient cycling beyond host–plant acquisition?* PGPR may have traits to solubilize and mobilize macronutrients and micronutrients. Some research into nitrogen and potassium root acquisition and soil leaching currently exists. More research is needed into micronutrients important to plant productivity and organic matter persistence, such as iron and manganese.
2. *How do PGPR affect organic matter persistence?* PGPR may shift relative abundance or activity of key players in the resident microbiome. These changes in microbe–microbe interactions could cascade to changes in soil properties mediated by microbes such as pH, erodibility, porosity, and water holding capacity of soil. Experimental applications of PGPR under field conditions that quantify soil parameters are needed.
3. *What are the multi‐trophic consequences of introduced PGPR?* PGPR are known to release volatile organic compounds (VOCs) that can deter herbivores. Some VOCs attract pollinators, but whether PGRP VOCs attract pollinators is unexplored. Indirect multitrophic interactions, mediated by changes in plant phenology like budbreak or flowering, may be spurred by PGPR and this remains a current knowledge gap. Additionally, PGPR may indirectly affect resident microbial abundances through antagonisms with bacterivores or fungivores.
4. *Do PGPR spread to other plant hosts in the ecosystem?* Host specificity is low among biostimulant microbes due to their application across various crops. Thus, escape from crops to adjacent ecosystems has a high potential, although persistence may be negligible. Mitigating effects caused by the escape of biostimulant microbes is an area of active research (Jack *et al*., 2020; Kim & Lee, 2020; Stirling & Silver, 2020; Ke *et al*., 2021), but the likelihood and frequency that PGPR escape occurs is uncertain.
5. *How long do microbial inoculants persist in the microbiome?* Persistence of biostimulant microbes is hypothesized to be short‐term, in the order of weeks following introductions. This question has been addressed in laboratory or glasshouse studies, but persistence after field applications remains a knowledge gap. When natural soils were inoculated in the laboratory with *Escherichia coli* the bacteria persisted for < 28 days (Mallon *et al*., 2018). The amount of time an inoculant may persist will depend on their traits. Traits conferring root colonization or biofilm formation (i.e. niche construction) will promote environment suitability for the inoculant, as well as traits for abiotic stress tolerance such as sporulation in bacteria or melanized cell walls in fungi. Biotic interactions will affect persistence and the ability to outcompete resident microbes for resources or to form positive interactions with resident microbes will enable the inoculants to establish. Taking a trait‐based microbial ecology perspective could aid researchers in predicting persistence of biostimulant microbes.
6. *How long do legacy effects of PGPR inoculants last?* After the inoculant ceases to persist in the community, they may have lasting effects on community composition and function (Mallon *et al*., 2018). Longitudinal studies that extend longer than the PGPR can be detected in the system are needed to address this knowledge gap. Changes in overall community function have been detected for weeks after an inoculant ceases to be detectable. It is possible the effects could last months or to the following growing season if the PGPR has caused community function to shift to an alternative stable state.
7. *Does horizontal gene transfer by PGPR lead to changes in overall ecosystem function?* Ecosystem functions like nutrient cycling are emergent properties of individual microbial functions. If PGPR act as mobile genetic element donors, they could affect the total genetic capacity of the microbial community for ecosystem processes. More studies that focus on mobile genetic elements of PGPR and the likelihood they are incorporated into the resident microbiome are needed. The predictability of horizontal gene transfer from PGPR to resident bacteria and whether it has lasting effects for community function warrants further experimentation.

## Ecosystem consequences of PGPR introductions

Biostimulant PGPR can have direct or indirect effects on ecosystem function (Fig. 1). Nutrient cycling can be altered directly by PGPR as they solubilize macronutrients, stimulate root nutrient uptake, and increase foliar nutrient content. Species of *Bacillus*, *Pseudomonas*, *Aspergillus*, and *Flavobacterium* produce phosphatases or organic acids that solubilize phosphorus from inorganic sources (De Pascale *et al*., 2018). Other PGPR can solubilize potassium (Han & Lee, 2005) and iron (Colla *et al*., 2015). Further, PGPR can indirectly influence solubility of macronutrients through shifting resident microbial interactions and community structure to a new state which could cascade to alter overall nutrient cycling (Nassal *et al*., 2018). Nutrient cycling is also modified as PGPR interact with roots. Nutrient uptake from organic fertilizers by roots can be stimulated and nutrient leaching from soil can be reduced by PGPR (Paungfoo‐Lonhienne *et al*., 2019). With increased nutrient availability in soil and translocation by roots, foliar chemistry can concomitantly shift toward nitrogen enrichment (Larimi *et al*., 2014). Understanding the extent to which other soil macronutrient and micronutrient availability can be directly or indirectly increased by PGPR or their interactions with the resident community will be important to harness the potential for PGPR used as biofertilizers.

Crop yields depend on nutrient availability as well as interactions with invertebrates. The impact of PGPR on pollinating invertebrates are largely unknown. Plant–microbe interactions can expand the growing season for a crop (Panke‐Buisse *et al*., 2015), thus influencing the timing of resource availability for migrating pollinators and birds that feed on fruits, seeds, and pollinating insects. We postulate that as plants gain greater access to nutrients, the quality and quantity of nectar and pollen may subsequently increase and possibly increase pollinator abundance. Alternatively, plants interacting with PGPR may release larger volumes of volatile organic compounds (VOCs) that attract pollinators (Liu & Brettell, 2019). PGPR can also mediate plant–herbivore interactions. PGPR release VOCs that reduce herbivory by invertebrates (Mohanty *et al*., 2021). Induced systemic plant defenses against herbivores are triggered by PGPR activating the jasmonic acid immune signaling pathway (Hol *et al*., 2013). One reason we lack information about biostimulant PGPR effects on pollinators is that much PGPR research is glasshouse‐based. Conducting field‐based pollinator studies with controlled PGPR inoculations will elucidate how these microbes may affect pollinators. In this way, plant–pollinator interactions and the ecosystem service of pollination could potentially be altered by the introduction of novel microbes.

Another ecosystem consequence that affects crop yield is plant disease suppression. Many PGPR in biostimulants are added to crops for the purposes of reducing pathogen loads (Pieterse *et al*., 2014). For example, the commercial biostimulant Companion^®^ Liquid Biological Fungicide (Douglas Plant Health, Liberty, MO, USA) contains *Bacillus velezensis* GB03. Application is intended to reduce pathogens such as those causing blight (*Phytophthora*, *Sclerotinia*), root rot (*Pythium*), and wilt (*Fusarium*). The hypothesized mechanism is that *B*. *velezensis* induces a systemic immune response across a wide range of plant hosts. Another potential mechanism is that the PGPR increases anti‐fungal indigenous taxa (Xiong *et al*., 2017). Predictions of plant productivity following PGPR application may improve if we work to understand how plant disease suppression is related to interactions between PGPR and resident communities.

Microbial interactions affect organic matter persistence. Biostimulant PGPR may have indirect effects on ecosystem functions such as the accumulation and decomposition of organic matter through their interactions with the resident microbiome (Hellequin *et al*., 2019), with plants, or their individual activity. It is not well understood how PGPR may interact with key indigenous decomposer fungi, thus potentially altering decomposition rates and the release of plant‐available nutrients (Kyker‐Snowman *et al*., 2020). Interactions with plant roots may also contribute to organic matter accumulation. Metabolites, VOCs, or auxins released by PGPR can affect root architecture, growth, and exudate production (Grover *et al*., 2021). These PGPR–root interactions may scale up to increase organic matter persistence, but more research is needed. PGPR may also contribute to soil organic matter accumulation through exuding biosynthesized compounds, such as extracellular enzymes, which can be long‐lived in soil and sorb to minerals to produce mineral‐associated organic matter (MaOM; Cotrufo *et al*., 2013). The interactions between PGPR microbes, plants, and resident fungi may have implications for rates of soil organic matter accumulation and decomposition.

## Legacy effects of PGPR introductions

Introduction of new microbes could lead to genetic changes in the inoculant or community, yet the genetic impact of PGPR introductions remains largely unexplored. Any location with a high microbial density, such as the rhizosphere, can support rapid and pervasive horizontal gene transfer (Kent *et al*., 2020; Yaffe & Relman, 2020). The introduction of new microbial species provides additional bidirectional transfer opportunities, with unpredictable consequences. For example, an introduced microbe could transfer its mobile genetic element to the resident community and thereby change existing plant–microbe interactions (Sullivan *et al*., 1995; Ling *et al*., 2016). Notably, these changes could persist even if the original inoculant were eliminated from the community. Alternately, an introduced microbe could acquire DNA from the resident community, potentially altering the function and persistence of the inoculant (Frazão *et al*., 2019; Munck *et al*., 2020). Genetic interactions between inoculant and resident microbial species remain largely unpredictable in natural environments and may have long‐lasting effects.

Legacy effects on resident microbial community structure can arise as PGPR establish and persist following introduction. Establishment and persistence can occur through at least two mechanisms: augmentation and displacement (Kurkjian *et al*., 2021). The resident microbial community may be augmented to include the inoculant such that no resident taxa are lost from the community. While resident presence may be unchanged, their relative abundance, function, and interactions with other members may be altered. These potential outcomes of augmentation need to be further researched, especially under glasshouse or field conditions with diverse resident microbiomes. Displacement of resident taxa and, thus, changes in composition and structure are also possible following PGPR introductions to the community depending on inoculant abundance and frequency of introductions (Albright *et al*., 2020). PGPR displace other taxa as they, for example, outcompete resident microbes for resources or through interference competition via antimicrobial compounds. Displacement is predicted to be more common than augmentation and the likelihood of either outcome seems unrelated to the number of cells introduced (i.e. propagule pressure; Kurkjian *et al*., 2021). In either case, the persistence of PGPR has the potential to alter community function as PGPR interact with and change abundance of resident taxa (Kalam *et al*., 2017).

Plant growth promoting rhizobacteria that persist following introduction can leave a legacy by altering their microenvironment to promote their own fitness, through a process known as ‘niche construction’ (Callahan *et al*., 2014; McNally & Brown, 2015). Microbes living in soil alter their microenvironment by changing resource availability, pH, and soil structure through their activity and interactions (Thakur & Wright, 2017). Introduction of a new PGPR strain runs the risk of triggering an environmental change via niche construction that can then lead to further changes in community composition (Suez *et al*., 2018) and potentially alter ecosystem functions.

Plant growth promoting rhizobacteria introductions that do not persist in the community may still leave legacy effects (Mallon *et al*., 2018; Kurkjian *et al*., 2021). Resident microbial species respond to introduced competitors by shifting their realized niche breadth, and the degree to which niche breadth shifts depends on diversity of the resident microbiome. Less diverse resident microbiomes respond to inoculants by shifting their niche breadth more than more diverse resident microbiomes (Mallon *et al*., 2018). Inoculants may have legacy effects if they alter the abundance of key functional groups, change trait distributions or interaction networks (da Costa *et al*., 2020), or act as a resource that can stimulate growth of functional groups (Wei *et al*., 2015; Kurkjian *et al*., 2021). Even transient inoculants have been shown to induce shifts in resident microbial communities by altering the abiotic environment or disrupting species interactions and abundances (Amor *et al*., 2020). Transient inoculants may alter the resident community diversity and niche structure resulting in long‐lasting consequences for plant health and soil processes. The legacies of introduced PGPR have received less attention than measuring the targeted outcome of plant growth, but understanding their legacies are critical to predicting and mitigating potential long‐lasting effects.

## A call for further research

Introducing PGPR may have unintended effects that cascade throughout the resident microbiome, adjacent plant communities, and the whole ecosystem (Box 1). We urge more research to elucidate how introducing PGPR impacts resident community structure and function, ecosystem function within the area of application (e.g. cropland), and the adjacent ecosystems. More research is needed on trophic cascades that microbial introductions may cause, especially those that could affect pollinator communities or soil organic matter persistence and thus directly influence plant health and productivity. The persistence of introduced PGPR over time as well as their legacy effects remain largely unknown. Engineering PGPR to persist longer may increase their effects for plant growth or health and this synthetic biology research is in progress (Haskett *et al*., 2021). Legacy effects may persist despite the introduced PGPR not remaining in the community. Longitudinal studies of introduced PGPR that measure microbial and ecosystem functions are needed. Our ability to predict establishment success of PGPR and the consequences for the resident microbiome, other trophic levels, and overall ecosystem function remain knowledge gaps in the rapidly expanding field of applied plant and microbial ecology.

## Author contributions

JAMM wrote the initial draft of this manuscript and conceived original ideas. JAMM, PEA, JKM, WM and MAC contributed to idea refinement, writing, and revising the manuscript.

## Data Availability

Data sharing is not applicable to this article as no new data were created or analyzed in this study.

## References

[nph18010-bib-0040] Albright MBN , Sevanto S , Gallegos‐Graves LV , Dunbar J . 2020. Biotic interactions are more important than propagule pressure in microbial community invasions. mBio 11: e02089‐20. 10.1128/mBio.02089-20 PMC7593967 33109758

[nph18010-bib-0001] Amor DR , Ratzke C , Gore J . 2020. Transient inoculants can induce shifts between alternative stable states of microbial communities. Science Advances 6: eaay8676. 32128414 10.1126/sciadv.aay8676 PMC7030923

[nph18010-bib-0002] Brown P , Saa S . 2015. Biostimulants in agriculture. Frontiers in Plant Science 6: 671. 26379695 10.3389/fpls.2015.00671 PMC4550782

[nph18010-bib-0003] Callahan BJ , Fukami T , Fisher DS . 2014. Rapid evolution of adaptive niche construction in experimental microbial populations. Evolution 68: 3307–3316. 25138718 10.1111/evo.12512

[nph18010-bib-0004] Colla G , Rouphael Y , Di Mattia E , El‐Nakhel C , Cardarelli M . 2015. Co‐inoculation of *Glomus* *intraradices* and *Trichoderma* *atroviride* acts as a biostimulant to promote growth, yield and nutrient uptake of vegetable crops. Journal of the Science of Food and Agriculture 95: 1706–1715. 25123953 10.1002/jsfa.6875

[nph18010-bib-0005] da Costa PB , van Elsas JD , Mallon C , dos Anjos Borges LG , Pereira Passaglia LM . 2020. Efficiency of probiotic traits in plant inoculation is determined by environmental constrains. Soil Biology and Biochemistry 148: 107893.

[nph18010-bib-0006] Cotrufo MF , Wallenstein MD , Boot CM , Denef K , Paul E . 2013. The Microbial Efficiency‐Matrix Stabilization (MEMS) framework integrates plant litter decomposition with soil organic matter stabilization: do labile plant inputs form stable soil organic matter? Global Change Biology 19: 988–995. 23504877 10.1111/gcb.12113

[nph18010-bib-0007] De Pascale S , Rouphael Y , Colla G . 2018. Plant biostimulants: innovative tool for enhancing plant nutrition in organic farming. European Journal of Horticultural Science 82: 1611–4434.

[nph18010-bib-0008] Deng S , Wipf HML , Pierroz G , Raab TK , Khanna R , Coleman‐Derr D . 2019. A plant growth‐promoting microbial soil amendment dynamically alters the strawberry root bacterial microbiome. Scientific Reports 9: 17677. 31776356 10.1038/s41598-019-53623-2 PMC6881409

[nph18010-bib-0009] Frazão N , Sousa A , Lässig M , Gordo I . 2019. Horizontal gene transfer overrides mutation in *Escherichia coli* colonizing the mammalian gut. Proceedings of the National Academy of Sciences, USA 116: 17906–17915. 10.1073/pnas.1906958116 PMC6731689 31431529

[nph18010-bib-0010] Grover M , Bodhankar S , Sharma A , Sharma P , Singh J , Nain L . 2021. PGPR mediated alterations in root traits: way toward sustainable crop production. Frontiers in Sustainable Food Systems 4: 112.

[nph18010-bib-0011] Han H , Lee K . 2005. Physiological responses of soybean‐inoculation of *Bradyrhizobium* *japonicum* with PGPR in saline soil conditions. Research Journal of Agriculture and Biological Sciences 1: 216–221.

[nph18010-bib-0039] Haskett TL , Tkacz A , Poole PS . 2021. Engineering rhizobacteria for sustainable agriculture. The ISME Journal 15: 949–964. 33230265 10.1038/s41396-020-00835-4 PMC8114929

[nph18010-bib-0012] Hellequin E , Monard C , Quaiser A , Henriot M , Klarzynski O , Binet F . 2019. Specific recruitment of soil bacteria and fungi decomposers following a biostimulant application increased crop residues mineralization. PLoS ONE 13: e0209089. 10.1371/journal.pone.0209089 PMC6312294 30596675

[nph18010-bib-0013] Hol WHG , Bezemer TM , Biere A . 2013. Getting the ecology into interactions between plants and the plant growth‐promoting bacterium *Pseudomonas* *fluorescens* . Frontiers in Plant Science 4: 81. 23596447 10.3389/fpls.2013.00081 PMC3622252

[nph18010-bib-0014] Jack CN , Petipas RH , Cheeke TE , Rowland JL , Friesen ML . 2020. Microbial inoculants: silver bullet or microbial Jurassic Park? Trends in Microbiology 29: 299–308. 33309525 10.1016/j.tim.2020.11.006

[nph18010-bib-0015] Kalam S , Das SN , Basu A , Podile AR . 2017. Population densities of indigenous *Acidobacteria* change in the presence of plant growth promoting rhizobacteria (PGPR) in rhizosphere. Journal of Basic Microbiology 57: 376–385. 28397264 10.1002/jobm.201600588

[nph18010-bib-0016] Ke J , Wang B , Yoshikuni Y . 2021. Microbiome engineering: synthetic biology of plant‐associated microbiomes in sustainable agriculture. Trends in Biotechnology 39: 244–261. 32800605 10.1016/j.tibtech.2020.07.008

[nph18010-bib-0017] Kent AG , Vill AC , Shi Q , Satlin MJ , Brito IL . 2020. Widespread transfer of mobile antibiotic resistance genes within individual gut microbiomes revealed through bacterial Hi‐C. Nature Communications 11: 1–9. 10.1038/s41467-020-18164-7 PMC7463002 32873785

[nph18010-bib-0018] Kurkjian HM , Akbari MJ , Momeni B . 2021. The impact of interactions on invasion and colonization resistance in microbial communities. PLoS Computational Biology 17: e1008643. 33481772 10.1371/journal.pcbi.1008643 PMC7857599

[nph18010-bib-0019] Kyker‐Snowman E , Wieder WR , Frey SD , Grandy AS . 2020. Stoichiometrically coupled carbon and nitrogen cycling in the MIcrobial‐MIneral carbon stabilization model v.1.0 (MIMICS‐CN v.1.0). Geoscientific Model Development 13: 4413–4434.

[nph18010-bib-0020] Larimi SB , Shakiba M , Mohammadinasab AD , Vahed M . 2014. Changes in nitrogen and chlorophyll density and leaf area of sweet basil (*Ocimum* *basilicum* L.) affected by biofertilizer and nitrogen application. International Journal of Biosciences 5: 256–265.

[nph18010-bib-0021] Ling J , Wang H , Wu P , Li T , Tang Y , Naseer N , Zheng H , Masson‐Boivin C , Zhong Z , Zhu J . 2016. Plant nodulation inducers enhance horizontal gene transfer of *Azorhizobium* *caulinodans* symbiosis island. Proceedings of the National Academy of Sciences, USA 113: 13875–13880. 10.1073/pnas.1615121113 PMC5137767 27849579

[nph18010-bib-0022] Liu H , Brettell LE . 2019. Plant defense by VOC‐induced microbial priming. Trends in Plant Science 24: 187–189. 30738790 10.1016/j.tplants.2019.01.008

[nph18010-bib-0023] Mallon CA , Le Roux X , van Doorn GS , Dini‐Andreote F , Poly F , Salles JF . 2018. The impact of failure: unsuccessful bacterial invasions steer the soil microbial community away from the inoculant's niche. The ISME Journal 12: 728–741. 29374268 10.1038/s41396-017-0003-y PMC5864238

[nph18010-bib-0024] McNally L , Brown SP . 2015. Building the microbiome in health and disease: niche construction and social conflict in bacteria. Philosophical Transactions of the Royal Society of London. Series B: Biological Sciences 370: 20140298. 26150664 10.1098/rstb.2014.0298 PMC4528496

[nph18010-bib-0025] Mohanty P , Singh PK , Chakraborty D , Mishra S , Pattnaik R . 2021. Insight into the role of PGPR in sustainable agriculture and environment. Frontiers in Sustainable Food Systems 5: 667150.

[nph18010-bib-0026] Munck C , Sheth RU , Freedberg DE , Wang HH . 2020. Recording mobile DNA in the gut microbiota using an *Escherichia coli* CRISPR‐Cas spacer acquisition platform. Nature Communications 11: 1–11. 10.1038/s41467-019-14012-5 PMC6946703 31911609

[nph18010-bib-0027] Nassal D , Spohn M , Eltlbany N , Jacquiod S , Smalla K , Marhan S , Kandeler E . 2018. Effects of phosphorus‐mobilizing bacteria on tomato growth and soil microbial activity. Plant and Soil 427: 17–37.

[nph18010-bib-0028] Panke‐Buisse K , Poole AC , Goodrich JK , Ley RE , Kao‐Kniffin J . 2015. Selection on soil microbiomes reveals reproducible impacts on plant function. The ISME Journal 9: 980–989. 25350154 10.1038/ismej.2014.196 PMC4817706

[nph18010-bib-0029] Paungfoo‐Lonhienne C , Redding M , Pratt C , Wang W . 2019. Plant growth promoting rhizobacteria increase the efficiency of fertilisers while reducing nitrogen loss. Journal of Environmental Management 233: 337–341. 30590263 10.1016/j.jenvman.2018.12.052

[nph18010-bib-0030] Pieterse CM , Zamioudis C , Berendsen RL , Weller DM , Van Wees SC , Bakker PA . 2014. Induced systemic resistance by beneficial microbes. Annual Review of Phytopathology 52: 347–375. 10.1146/annurev-phyto-082712-102340 24906124

[nph18010-bib-0031] Stirling F , Silver PA . 2020. Controlling the implementation of transgenic microbes: are we ready for what synthetic biology has to offer. Molecular Cell 78: 614–623. 32442504 10.1016/j.molcel.2020.03.034 PMC7307494

[nph18010-bib-0032] Suez J , Zmora N , Zilberman‐Schapira G , Mor U , Dori‐Bachash M , Bashiardes S , Zur M , Regev‐Lehavi D , Ben‐Zeev Brik R , Federici S *et al*. 2018. Post‐antibiotic gut mucosal microbiome reconstitution is impaired by probiotics and improved by autologous FMT. Cell 174: 1406–1423. e1416. 30193113 10.1016/j.cell.2018.08.047

[nph18010-bib-0033] Sullivan JT , Patrick HN , Lowther WL , Scott DB , Ronson CW . 1995. Nodulating strains of *Rhizobium loti* arise through chromosomal symbiotic gene transfer in the environment. Proceedings of the National Academy of Sciences, USA 92: 8985–8989. 10.1073/pnas.92.19.8985 PMC41092 7568057

[nph18010-bib-0034] Thakur MP , Wright AJ . 2017. Environmental filtering, niche construction, and trait variability: the missing discussion. Trends in Ecology & Evolution 32: 884–886. 29102055 10.1016/j.tree.2017.09.014

[nph18010-bib-0035] Wei Z , Yang T , Friman V‐P , Xu Y , Shen Q , Jousset A . 2015. Trophic network architecture of root‐associated bacterial communities determines pathogen invasion and plant health. Nature Communications 6: 8413. 10.1038/ncomms9413 PMC4598729 26400552

[nph18010-bib-0036] Xiong W , Guo S , Jousset A , Zhao Q , Wu H , Li R , Kowalchuk GA , Shen Q . 2017. Bio‐fertilizer application induces soil suppressiveness against *Fusarium* wilt disease by reshaping the soil microbiome. Soil Biology and Biochemistry 114: 238–247.

[nph18010-bib-0037] Yaffe E , Relman DA . 2020. Tracking microbial evolution in the human gut using Hi‐C reveals extensive horizontal gene transfer, persistence and adaptation. Nature Microbiology 5: 343–353. 10.1038/s41564-019-0625-0 PMC6992475 31873203

[nph18010-bib-0038] Zheng W , Zeng S , Bais H , LaManna JM , Hussey DS , Jacobson DL , Jin Y . 2018. Plant growth‐promoting rhizobacteria (PGPR) reduce evaporation and increase soil water retention. Water Resources Research 54: 3673–3687.

